# Hesperetin Attenuates Liver Fibrosis in Mice by Modulating Selected Gut Microbial Populations and SIRT2-Associated Inflammatory Signaling

**DOI:** 10.5812/ijpr-170978

**Published:** 2026-06-17

**Authors:** Anzhao Wu, Lingjie Xin, Yuji Liang, Luyan Sun, Qian Liu, Bin Wang, YaPing Li, Yuehong Cui, Umar Saeed, Bo Yang, Zahra Zahid Piracha

**Affiliations:** 1 Department of Integrated Traditional Chinese and Western Medicine, Public Health Clinical Center Affiliated to Shandong University, Jinan, China; 2 Department of Pharmacotoxicology, School of Pharmacy, Shandong First Medical University, Jinan, China; 3 Second Department of Hepatology, Public Health Clinical Center Affiliated to Shandong University, Jinan, China; 4 School of Nursing, Shandong University of Traditional Chinese Medicine, Jinan, China; 5 Clinical and Biomedical Research Center (CBRC), Foundation University Islamabad (FUI), Islamabad, Pakistan; 6 Korea University College of Health Sciences, Korea University, Seoul, South Korea; 7 Széchenyi István University, Győr, Hungary; 8 Department of Medical Research, International Center of Medical Sciences Research (ICMSR), Islamabad, Pakistan; 9 International Center of Medical Sciences Research (ICMSR), Essex, United Kingdom

**Keywords:** Hesperetin, Liver Fibrosis, CCl_4_, Sirius Red Staining, ALT, AST, SIRT2, Gut Microbiota, Inflammation

## Abstract

**Background:**

Chronic liver injury progressively induces fibrotic remodeling through persistent inflammatory activity and aberrant extracellular matrix accumulation. Growing evidence indicates that this process involves not only intrahepatic signaling but also gut–liver crosstalk and sirtuin-regulated inflammatory pathways. Hesperetin, a citrus-derived flavanone with reported antioxidant and anti-inflammatory properties, has not been adequately evaluated with respect to microbiota-associated changes and SIRT2-associated signaling during fibrotic liver injury.

**Objectives:**

This study investigated whether hesperetin attenuates CCl_4_-induced liver fibrosis in mice by modulating selected gut microbial populations and suppressing SIRT2-associated inflammatory signaling pathways.

**Methods:**

Male mice were randomly assigned to the vehicle, CCl_4_, and CCl_4_ + hesperetin groups (n = 5 per group). Liver fibrosis was induced by intraperitoneal administration of CCl_4_ twice weekly for six weeks, and hesperetin was administered orally at 100 mg/kg/day. Histological, biochemical, inflammatory, SIRT2-associated, and selected gut microbial endpoints were assessed. The expression levels of α-SMA, TGF-β1, TNF-α, IL-6, and SIRT2 were quantitatively analyzed using quantitative polymerase chain reaction (qPCR), Western blotting, and an enzyme-linked immunosorbent assay (ELISA). Levels of selected gut microflora were measured using targeted qPCR for Firmicutes, *Bacteroidetes*, Akkermansia, and *Escherichia coli*.

**Results:**

CCl_4_ exposure increased collagen deposition, collagen proportionate area (CPA), and serum alanine aminotransferase (ALT) and aspartate aminotransferase (AST) activities, indicating fibrotic remodeling and hepatocellular injury. Hesperetin treatment significantly decreased collagen deposition and CPA and partially normalized ALT and AST activities compared with CCl_4_ treatment alone. Fibrosis biomarkers, including α-SMA and TGF-β1, and inflammatory cytokines, including TNF-α and IL-6, showed significant decreases in expression at both the mRNA and protein levels. Hesperetin reduced CCl_4_-associated SIRT2 upregulation and restored acetyl-α-tubulin levels, suggesting attenuation of SIRT2-associated deacetylase activity.

**Conclusions:**

These exploratory findings suggest that hesperetin attenuates CCl_4_-induced liver fibrosis by reducing collagen accumulation and biochemical liver injury, suppressing inflammatory and fibrogenic signaling, modulating selected gut microbial populations, and regulating SIRT2-associated acetylation signaling.

## 1. Background

Liver fibrosis is a pathological wound-healing response to chronic liver injury caused by viral hepatitis, excessive alcohol intake, nonalcoholic steatohepatitis (NASH), autoimmune hepatitis, and toxic agents such as carbon tetrachloride (CCl_4_). It remains a major clinical challenge worldwide because progressive fibrosis can ultimately lead to cirrhosis, liver failure, and hepatocellular carcinoma (1, 2). Liver fibrosis is characterized by excessive deposition of extracellular matrix (ECM) proteins, particularly collagen I and III. Ultimately, these changes alter liver architecture, sinusoidal structure, and overall hepatic function (3, 4). Unless the underlying etiology of fibrosis is treated, progression to cirrhosis may result in catastrophic outcomes, including liver failure and hepatocellular carcinoma (5, 6). Although considerable progress has been made in treating etiologies through antiviral therapy and management of metabolic syndrome, to date, no drugs are approved for treating fibrosis; therefore, new antifibrotic therapies must be developed to target central regulatory pathways of inflammation (7).

Fibrogenesis is regulated by complex cellular and molecular mechanisms that include immunological processes, cellular injury, and stromal remodeling (8). Hepatic stellate cells are considered central effectors of liver fibrosis, as their activation into myofibroblast-like cells under profibrotic stimuli, such as TGF-β1, promotes collagen deposition in the extracellular matrix, thereby exacerbating fibrosis, along with other proteins such as contractile factors (9). Hepatic stellate cell (HSC) activation is strongly influenced by the inflammatory microenvironment. Proinflammatory factors such as tumor necrosis factor-alpha (TNF-α) and interleukin-6 (IL-6) may promote chronic inflammatory responses, enhancing sustained recruitment and activation of inflammatory cells and thereby perpetuating the injury cascade in the liver, culminating in scarring (10). Thus, therapeutic strategies that can regulate these inflammatory cytokines while curbing HSC activation may be advantageous for resolving liver fibrosis, as they simultaneously counter two key pathways in fibrogenesis.

Beyond intrahepatic factors, emerging evidence suggests that the gut-liver axis may play a crucial role in the pathogenesis and progression of chronic liver disease (CLD) by modulating microbial composition, intestinal barrier function, and immune responses (11). Among dietary factors, those affecting the gut environment, such as fiber, prebiotics, and bacterial exopolysaccharides, can alter the metabolic activities of gut microbes and modulate host inflammatory responses, with important implications for the degree of liver injury (11). Microbiota-directed interventions, including probiotics, prebiotics, and dietary bioactives, have therefore attracted attention as potential strategies to regulate gut microbial composition and host inflammatory responses (12, 13). Under pathological conditions, such as intestinal dysbiosis and increased permeability, microbial products can be more readily released into the portal circulation and may stimulate inflammatory responses and exacerbate liver fibrosis. Consistent with its critical role in CLD pathogenesis, microbiome analyses of patients with cirrhosis have consistently documented disruptions in microbiota composition, including the predominance of pathogenic Enterobacteriaceae, such as *Escherichia coli*, and a decrease in certain microbiota associated with intestinal health and barrier function (14-16). Collectively, these findings support microbial homeostasis as a relevant therapeutic dimension in chronic liver pathology, particularly when inflammatory amplification is a central disease feature.

Sirtuins are NAD^+^-dependent deacetylases that integrate metabolic state with stress adaptation, inflammation control, and cellular remodeling (17, 18). SIRT2, originally characterized as an NAD^+^-dependent tubulin deacetylase, is predominantly cytoplasmic and has been implicated in cytoskeletal dynamics, oxidative stress regulation, and inflammatory signaling (19). More broadly, SIRT2 has been positioned as a multifunctional regulator in diverse physiological and pathological contexts, including disorders featuring inflammation and tissue remodeling (20). In liver disease, SIRT2 has been implicated in pathways that intersect inflammation and fibrogenesis, positioning it as a modifiable node connecting injury signals to downstream transcriptional and structural responses. Importantly, experimental evidence indicates that SIRT2 inhibition suppresses liver fibrosis, supporting the plausibility of SIRT2 as a therapeutic target in fibrotic liver injury models (21, 22). However, in the specific context of CCl_4_-driven fibrosis, the extent to which SIRT2 contributes to inflammatory amplification and fibrogenic progression, and whether it can be modulated by dietary bioactives, remains insufficiently clarified.

Flavonoids are polyphenolic compounds found in fruits and vegetables and have been extensively studied in relation to chronic diseases and tissue injury because of their antioxidant and anti-inflammatory properties (23, 24). Evidence from functional food and mechanistic studies suggests that phenolic compounds can regulate oxidative stress, inflammatory cytokine production, and microbial metabolism, thereby influencing host inflammatory profiles (23, 24). Flavanones in citrus fruits have attracted considerable interest because of their prevalence in the diet and multitarget biological activities. Methodologies for their measurement, and their associations with bioavailability and antioxidant activity, have been emphasized (25, 26). For example, hesperetin, a major citrus flavanone, has shown hepatoprotective activity in metabolic liver injury by attenuating hepatic lipid accumulation via AMPK-associated pathways (27). However, whether hesperetin protects the liver from toxin-induced fibrogenesis through mechanisms encompassing gut microbiota regulation and intracellular hepatic signaling modulators such as SIRT2 has not been clearly evaluated.

## 2. Objectives

In the present study, we evaluated the therapeutic effects of hesperetin in a murine model of CCl_4_-induced liver fibrosis. We assessed histological injury and fibrotic remodeling, as well as inflammatory markers and SIRT2-associated inflammatory signaling, to determine whether hesperetin suppresses inflammation-driven fibrogenesis. In parallel, we examined selected gut microbial populations represented by Firmicutes, *Bacteroidetes*, Akkermansia, and *Escherichia coli*, which are representative taxa indicative of microbial balance and dysbiosis in chronic liver disease (14-16). By integrating histopathology, biochemical and molecular readouts, and targeted microbial profiling, this work tests the hypothesis that hesperetin confers hepatoprotection through the coordinated regulation of gut microbial homeostasis and SIRT2-linked inflammatory pathways.

## 3. Methods

### 3.1. Animals and Experimental Design

Male BALB/c mice (6 - 8 weeks, 22 - 25 g) were obtained from the institutional animal facility and housed under controlled conditions (22 ± 2 °C, 12-h light/dark cycle), with free access to standard chow and water. All procedures followed institutional ethical guidelines and complied with the Animal Research: Reporting of in vivo Experiments (ARRIVE) recommendations for animal care, monitoring, and humane endpoints. Mice were randomly allocated to three groups (n = 5 per group), and treatments were administered according to a predefined dosing schedule:

1. Vehicle control: olive oil (intraperitoneal, i.p.) and 0.5% carboxymethyl cellulose (CMC; p.o.);

2. CCl_4_: CCl_4_ 1 mL/kg (diluted 1:3 in olive oil, i.p., twice weekly);

3. CCl_4_ + hesperetin: CCl_4_ as above plus hesperetin 100 mg/kg/day (administered orally, p.o.), suspended in 0.5% CMC.

Carbon tetrachloride (CCl_4_; Sigma-Aldrich, ≥99.5%, cat. 289116) was diluted 1:3 (v/v) in olive oil; mice received 0.25 mL/kg CCl_4_ (total injection volume 1 mL/kg) via intraperitoneal injection twice weekly for 6 weeks. Hesperetin (Sigma-Aldrich, ≥95%, cat. W431300) was suspended in 0.5% CMC and administered by oral gavage (10 mL/kg) once daily at 100 mg/kg. Treatments continued for 6 weeks. At the endpoint, mice were anesthetized and euthanized; blood and liver tissues were collected for analysis (28, 29).

### 3.2. Intervention and Exposure Details

CCl_4_ was prepared fresh for each dosing session by diluting the stock solution 1:3 (v/v) in olive oil and mixing thoroughly to obtain a uniform working solution before intraperitoneal injection. Mice received a total injection volume of 1 mL/kg, corresponding to 0.25 mL/kg CCl_4_. CCl_4_ injections were administered twice weekly on nonconsecutive days at approximately the same time of day throughout the 6-week protocol. Hesperetin was suspended in 0.5% carboxymethyl cellulose in distilled water, mixed thoroughly before administration, and delivered by oral gavage at 10 mL/kg using a standardized gavage procedure. On CCl_4_ injection days, hesperetin or vehicle was administered by oral gavage approximately 1 hour before the CCl_4_ injection, and this interval was kept consistent across all animals throughout the study. Vehicle-treated animals underwent the same handling and dosing schedule as the treatment groups.

### 3.3. Sample Size Justification

This study was designed as an exploratory pilot in vivo investigation to assess the potential antifibrotic effects of hesperetin in a CCl_4_-induced liver fibrosis model. A sample size of five mice per group was selected based on consistency with comparable exploratory liver fibrosis studies using histological, biochemical, and molecular endpoints, together with the ethical principle of minimizing animal use. No formal a priori power calculation was performed; therefore, this study should not be regarded as a definitive hypothesis-testing experiment. Instead, the results provide preliminary effect estimates and mechanistic signals that require confirmation in adequately powered studies. Accordingly, the findings should be interpreted as preliminary and hypothesis-generating, and they provide initial effect estimates to support future adequately powered studies. Predefined outcomes included collagen proportionate area, serum ALT and AST, fibrosis-related markers, inflammatory cytokines, and SIRT2-associated readouts.

### 3.4. Inclusion/Exclusion Criteria and Attrition

All animals allocated to the study were included in the final analysis. No animals died, were removed, or were excluded during the 6-week experimental period. All predefined endpoints (histological, biochemical, and molecular analyses) were assessed using samples obtained from all animals in each group. No missing data occurred, and no imputation or data replacement procedures were required.

### 3.5. Animal Welfare Monitoring and Humane Endpoints

Animal welfare was monitored daily throughout the 6-week experimental period, with attention to general health, behavior, and signs of distress. Body weight was measured at least twice weekly as an indicator of treatment-related effects and welfare concerns. Clinical signs were evaluated in terms of activity, posture, grooming behavior, hair condition, consumption of feed and water, and signs of pain/toxicity (lethargy, piloerection, immobility). Analgesic drugs were not routinely used, as no procedures were expected to produce pain exceeding that associated with the injection procedure itself; nevertheless, any evidence of pain or distress was carefully documented. Humane endpoints, including > 20% body weight loss, extreme lethargy, failure to access food/water, or other signs of distress, required euthanasia of the affected animals; none reached these endpoints during the experimental period.

### 3.6. Randomization, Blinding, and Experimental Unit

Following acclimatization, mice were randomized to groups using computer-generated random numbers. Individual mice served as the experimental units. Investigators responsible for histological assessment, collagen proportionate area measurements, biochemical assays, ELISA, qPCR analysis, and Western blot densitometry were blinded to group allocation using coded samples until completion of the primary analyses. Because treatment preparation and administration differed among groups, blinding during dosing was not possible.

### 3.7. Sirius Red Staining and Collagen Quantification

To determine the degree of liver fibrosis, adjacent paraffin liver sections were stained with a 0.1% solution of Sirius Red F3B in saturated picric acid for 1 hour. After rinsing with acidified water, sections were dried and mounted on slides. Collagen fibers stained red and were observed under a light microscope at approximately 100× total magnification (10× objective). Collagen proportionate area (CPA) was quantified using ImageJ software and expressed as the percentage of Sirius Red-stained area relative to the total area. For each animal, several independent areas (3 areas per section) were photographed from at least two independent liver sections obtained from comparable regions of the organ. Sampling was performed using systematic randomization to avoid selection bias. A uniform color threshold was set to identify Sirius Red-stained areas and was held constant throughout the analysis.

### 3.8. Serum ALT and AST Assays

Blood samples were collected following sacrifice and allowed to clot; sera were then prepared by centrifugation and subjected to biochemical analyses. Serum ALT and AST activities were measured using kinetic colorimetric assay kits (Thermo Fisher Scientific; catalog number TR71121 for ALT and TR70121 for AST). Enzyme activities were calculated from absorbance changes per unit time (ΔA/min) and converted to U/L using the manufacturer-provided factor. For assay validation and run-to-run comparability, the recommended kit control/calibrator procedure was applied in each run, and all samples were analyzed under identical assay conditions, including reagent preparation and calculation settings.

### 3.9. RNA Isolation and Quantitative PCR

Total RNA was isolated from liver tissue using TRIzol reagent (Invitrogen), according to the manufacturer’s guidelines. RNA quality and quantity were assessed by NanoDrop analysis. Reverse transcription was performed with one microgram of total RNA using the cDNA synthesis kit (Thermo Fisher). SYBR Green Master Mix (Applied Biosystems) was used for qPCR on the StepOnePlus instrument. Primers specifically amplified genes encoding α-SMA (Acta2), TGF-β1, TNF-α, IL-6, SIRT2, and regions of gut microbiota 16S rRNA (Firmicutes, *Bacteroidetes*, Akkermansia, *E. coli*). GAPDH was used as an endogenous control for mRNA expression, whereas universal bacterial primers were used for normalization of bacterial gene expression. *Relative expression* was assessed using the 2^-ΔΔCt^ approach.

### 3.10. Western Blotting

Liver samples were homogenized in RIPA buffer containing a protease inhibitor cocktail. Protein concentration was measured by BCA assay. Equal amounts of protein (30 μg) were electrophoresed on 10 - 12% SDS-PAGE and transferred to a PVDF membrane (30, 31). The membrane was blocked with 5% BSA and probed overnight at 4 °C with primary antibodies targeting α-SMA (Abcam, cat. no. ab5694), TGF-β1 (Cell Signaling Technology, cat. no. 3711), TNF-α (Abcam, cat. no. ab6671), IL-6 (Cell Signaling Technology, cat. no. 12912), SIRT2 (Cell Signaling Technology, cat. no. 12650), acetyl-α-tubulin (K40) (Cell Signaling Technology, cat. no. 5335), and GAPDH (Cell Signaling Technology, cat. no. 5174). Membranes were then incubated with HRP-labeled secondary antibodies against rabbit IgG (Cell Signaling Technology, cat no. 7074) or mouse IgG (Cell Signaling Technology, cat no. 7076). Protein bands were visualized using ECL luminescence, and band density was measured using ImageJ software. Band densities were measured relative to GAPDH as a control. Densitometry was performed only on bands within the linear detection range, and exposure times were kept constant across all samples.

### 3.11. Enzyme-Linked Immunosorbent Assay

TNF-α and IL-6 protein concentrations in sera and liver homogenates were quantified using commercially available ELISA kits (R&D Systems, Minneapolis, MN, USA; Quantikine Mouse TNF-α ELISA kit, catalog number: MTA00B; Quantikine Mouse IL-6 ELISA kit, catalog number: M6000B) according to the manufacturer’s protocol. Optical density was determined at 450 nm, and protein concentrations were calculated from the standard curve. ELISA assays were performed on coded samples, and protein concentrations were quantified before the code was broken.

### 3.12. Gut Microbial Analysis by qPCR

Fresh fecal pellets were collected from each mouse before sacrifice. Microbial DNA was extracted using a QIAamp DNA Stool Mini Kit (Qiagen). qPCR amplification was performed using target-specific primers for Firmicutes, *Bacteroidetes*, Akkermansia muciniphila, *Escherichia coli*, and universal bacterial 16S rRNA, as listed in Table S1 in the Supplementary File. These taxa were selected a priori based on their reported relevance to gut–liver axis regulation, dysbiosis in liver fibrosis, and representation of beneficial (Firmicutes, Akkermansia) and potentially dysbiotic or pathogenic populations (*Bacteroidetes*, *Escherichia coli*). Amplicon size and primer sequences are provided in Table S1 in the Supplementary File. Data were normalized to universal bacterial 16S rRNA and expressed as relative abundance using the 2^-ΔΔCt^ method relative to the Vehicle group. All samples were analyzed in technical duplicates, and mean Ct values were used for downstream calculations (32).

### 3.13. Assessment of SIRT2 Activity

To evaluate the functional activity of SIRT2, acetylation of α-tubulin (K40) was used as a surrogate marker. Western blotting for acetyl-α-tubulin was performed as described above. Increased acetylation indicated reduced SIRT2 activity.

### 3.14. Statistical Analysis

Data are presented as mean ± SD. For each endpoint, the exact number of animals analyzed per group is indicated in the corresponding figure legend; unless otherwise stated, n = 5 animals per group. Statistical comparisons were performed using one-way ANOVA followed by Tukey’s multiple-comparison post hoc test in GraphPad Prism 9. Before ANOVA, data distributions were assessed for approximate normality and homogeneity of variance using standard software-based diagnostic checks. Because this was an exploratory multi-endpoint study, each outcome was analyzed within its own predefined biological domain rather than as part of a single pooled hypothesis family; therefore, Tukey’s post hoc correction was applied within each ANOVA model. Exact P values are reported where feasible, and effect magnitude was interpreted together with the consistency of changes across histological, biochemical, and molecular endpoints. For all statistical analyses, the individual mouse was defined as the experimental unit, and technical replicates were averaged to yield a single value per animal, thereby avoiding pseudoreplication.

## 4. Results

All animals completed the experimental protocol, and there were no exclusions, deaths, or missing endpoint data.

4.1. Sirius Red-Based Collagen Deposition and Serum Transaminases Confirm Antifibrotic and Hepatoprotective Effects of Hesperetin

**Figure 1.**
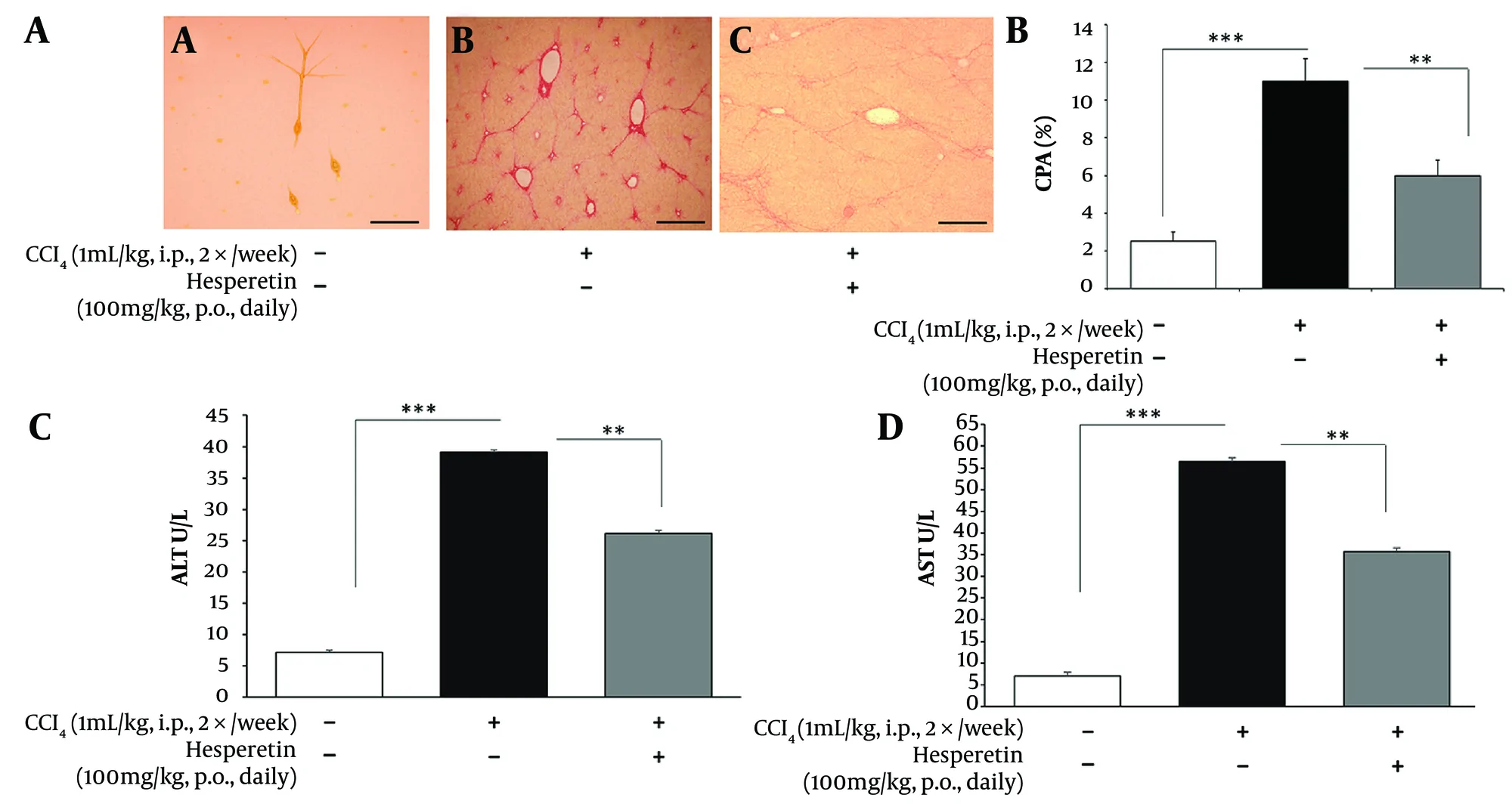
Sirius Red–based fibrosis assessment and serum transaminase activities. A, Sirius Red F3B staining (0.1% in saturated picric acid, 1 h) of 5 µm paraffin-embedded liver sections from Vehicle, CCl_4_, and CCl_4_ + hesperetin groups after 6 weeks to visualize fibrillar collagen deposition; images were acquired at 100× total magnification. Images are representative of at least three liver sections per animal acquired under identical magnification and imaging settings across groups. Scale bars = 100 μm. B, Fibrosis burden quantified as collagen proportionate area (CPA) from Sirius Red–positive regions using ImageJ and reported as the percentage of stained area relative to the total tissue area. C, Serum alanine aminotransferase (ALT) activity determined by a kinetic colorimetric method and presented in U/L. D, Serum aspartate aminotransferase (AST) activity determined by the same kinetic colorimetric method and presented in U/L. Values are shown as mean ± SD for five animals per group. Group differences were evaluated using one-way ANOVA with Tukey’s post hoc multiple-comparison test.

To evaluate the extent of CCl_4_-induced liver fibrosis and the protective effects of hesperetin, we examined representative Sirius Red-stained liver sections (Figure 1A) and quantified fibrosis severity using the collagen proportionate area (CPA; % Sirius Red-positive area) (Figure 1B). Vehicle-treated mice showed minimal collagen deposition, largely restricted to periportal regions. In contrast, CCl_4_ treatment produced marked collagen accumulation with prominent bridging fibrotic septa. Hesperetin co-treatment visibly reduced Sirius Red-positive staining, with thinner fibrotic septa and less extensive bridging than in the CCl_4_ group. Quantitative analysis supported these histological observations: mean CPA increased from 2.5% in the Vehicle group to 11.0% in the CCl_4_ group, corresponding to a mean difference of 8.5 percentage points, and decreased to 6.0% in the CCl_4_ + hesperetin group, representing a 5.0 percentage-point reduction versus CCl_4_ (Figure 1B).

To further assess hepatocellular injury, we measured serum alanine aminotransferase (ALT) and aspartate aminotransferase (AST) activities using a kinetic assay method (Figure 1C-D). ALT activity increased from 7.16 ± 0.39 U/L in the Vehicle group to 39.11 ± 0.39 U/L after CCl_4_ exposure, indicating marked hepatocellular injury. Hesperetin treatment reduced ALT to 26.19 ± 0.39 U/L, indicating partial biochemical protection compared with CCl_4_ treatment alone. Similarly, AST activity increased from 6.98 ± 0.87 U/L in Vehicle-treated mice to 56.40 ± 0.87 U/L in the CCl_4_ group and was reduced to 35.62 ± 0.87 U/L following hesperetin treatment. Because this was an exploratory study with a small sample size, these findings were interpreted based on the consistency and direction of biological effects rather than extremely small P values alone. Collectively, these findings indicate that hesperetin attenuates biochemical liver injury in parallel with reduced collagen deposition and lower CPA.

### 4.2. Hesperetin Reduces CCl_4_-Induced Expression of Fibrosis-Related Markers

**Figure 2.**
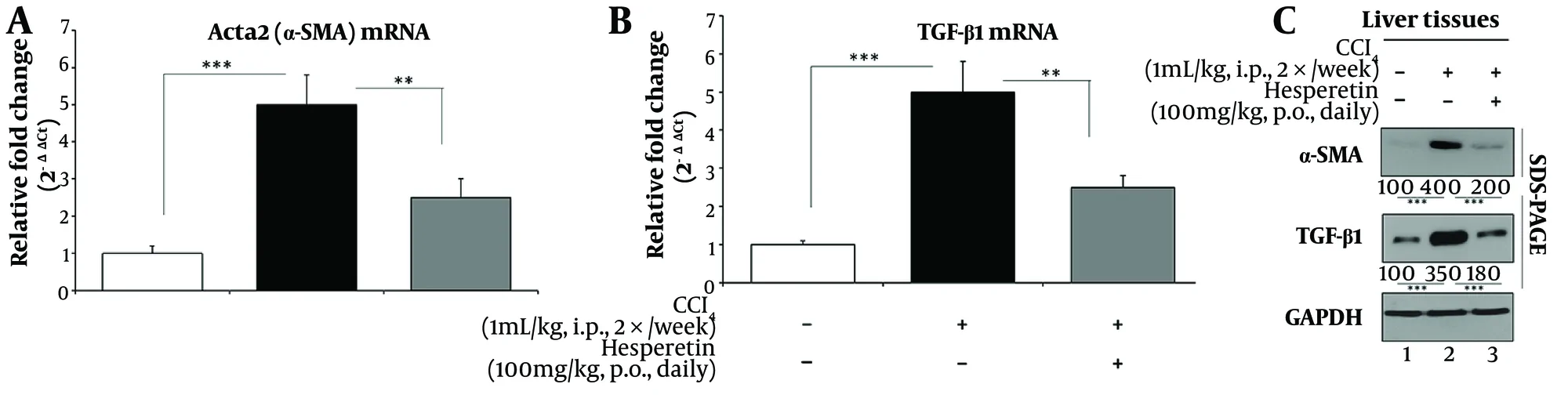
Hepatic fibrogenic marker expression. A, Hepatic α-smooth muscle actin (α-SMA/Acta2) transcript levels measured by qPCR, normalized to GAPDH, and expressed relative to the Vehicle group. B, Hepatic transforming growth factor-β1 (TGF-β1) mRNA levels measured by qPCR, normalized to GAPDH, and presented as fold change versus Vehicle. C, Immunoblot analysis of α-SMA and TGF-β1 in liver lysates (30 µg protein per lane), with GAPDH used as the reference control. Band signals were quantified in ImageJ, normalized to GAPDH, and reported relative to Vehicle (Vehicle = 100%). Data are expressed as mean ± SD (n = 5 per group). Statistical significance was tested by one-way ANOVA followed by Tukey’s multiple-comparison procedure.

To assess whether hesperetin influenced stellate cell activation, we measured α-SMA (Acta2) mRNA expression by qPCR (Figure 2A). Vehicle livers maintained baseline α-SMA expression (Vehicle = 1.0 ± 0.2 fold). CCl_4_ strongly induced α-SMA to 5.0 ± 1.2-fold relative to Vehicle (P < 0.001 vs Vehicle). Hesperetin significantly reduced this induction to 2.5 ± 0.6-fold relative to Vehicle (P < 0.01 vs CCl_4_), indicating partial suppression of stellate cell activation. To determine whether hesperetin suppressed profibrotic signaling, we measured TGF-β1 transcripts (Figure 2B). Vehicle livers expressed basal levels (Vehicle = 1.0 ± 0.2 fold). CCl_4_ induced TGF-β1 to 5.0 ± 1.5-fold relative to Vehicle (P < 0.001 vs Vehicle). Hesperetin reduced expression to 2.5 ± 0.7-fold relative to Vehicle (P < 0.01 vs CCl_4_), indicating its ability to blunt profibrotic transcription. To validate these findings at the protein level, we performed Western blotting. α-SMA and TGF-β1 bands were faint in Vehicle but markedly intensified in CCl_4_ (Figure 2C). Densitometry normalized to GAPDH revealed α-SMA levels of 400 ± 35% of Vehicle and TGF-β1 levels of 350 ± 30% of Vehicle (Vehicle = 100%; P < 0.001 vs Vehicle). Hesperetin treatment reduced α-SMA to 200 ± 25% of Vehicle and TGF-β1 to 180 ± 20% of Vehicle (P < 0.001 vs CCl_4_). Overall, these data demonstrate that hesperetin attenuates fibrogenesis by reducing stellate cell activation and suppressing TGF-β1 signaling at both transcriptional and protein levels.

4.3. Hesperetin Attenuates CCl_4_-Induced Inflammatory Responses in Mouse Liver by Reducing TNF-α and IL-6 Expression at Both Transcriptional and Protein Levels

**Figure 3.**
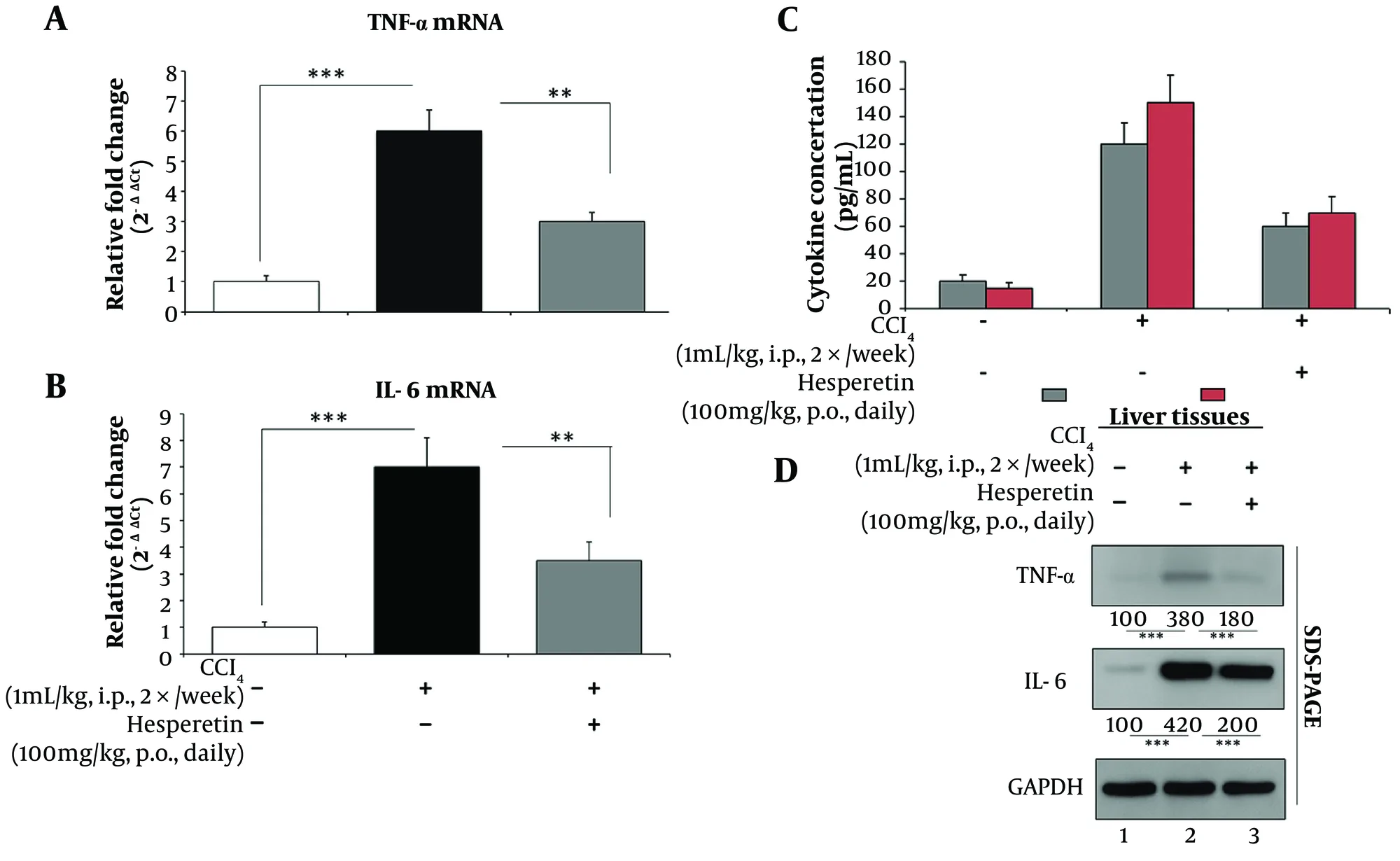
Proinflammatory cytokine analysis. A, Relative hepatic TNF-α mRNA expression assessed by qPCR after normalization to GAPDH. B, Relative hepatic IL-6 mRNA expression assessed by qPCR after normalization to GAPDH. C, Serum concentrations of TNF-α and IL-6 measured by ELISA and expressed in pg/mL. D, Immunoblot detection of TNF-α and IL-6 in liver homogenates, with GAPDH as the loading control; densitometric values were normalized to GAPDH and expressed relative to the Vehicle group (Vehicle = 100%). All values are presented as mean ± SD from five mice in each group. Comparisons among groups were performed using one-way ANOVA with Tukey’s post hoc test.

To assess hepatic inflammatory activation, we measured TNF-α and IL-6 expression by qPCR, ELISA, and Western blotting (Figure 3). Vehicle-treated mice showed low basal cytokine expression, whereas CCl_4_ markedly increased TNF-α and IL-6 at both transcript and protein levels. Hesperetin reduced these cytokine responses, supporting an anti-inflammatory effect in the CCl_4_-injured liver. ELISA analysis showed that CCl_4_ elevated serum TNF-α and IL-6 concentrations, whereas hesperetin partially reduced both cytokines compared with CCl_4_ alone. Western blot analysis showed a consistent pattern, with stronger TNF-α and IL-6 bands in the CCl_4_ group and reduced expression after hesperetin treatment. Collectively, these findings indicate that hesperetin attenuates CCl_4_-induced hepatic inflammation by reducing coordinated TNF-α and IL-6 responses at the transcript and protein levels.

### 4.4. Hesperetin Modulates Hepatic SIRT2 Expression and Activity in CCl_4_-Induced Liver Fibrosis

**Figure 4.**
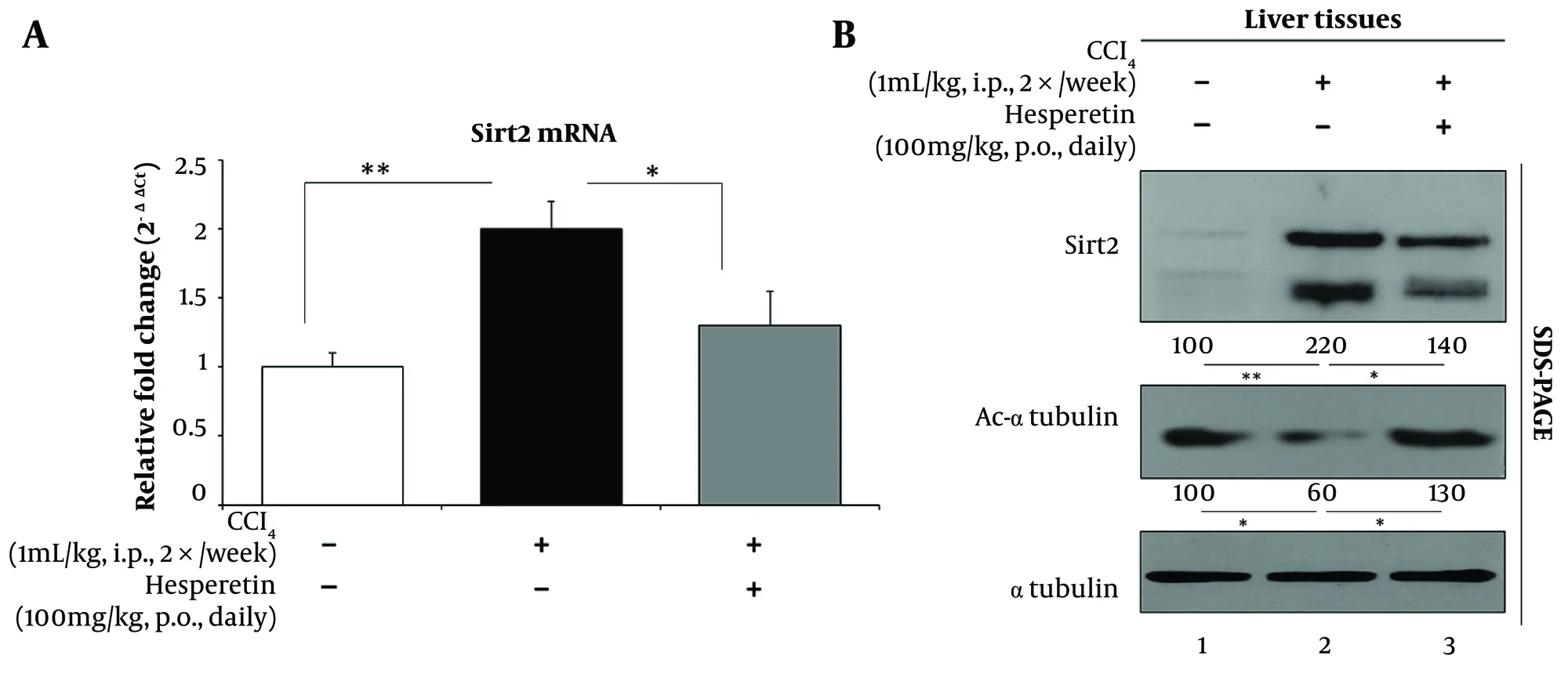
Hepatic SIRT2 expression and activity. A, Hepatic SIRT2 mRNA abundance measured by qPCR, normalized to GAPDH, and expressed relative to Vehicle. B, Western blot detection of SIRT2 protein in liver samples (30 µg per lane), using α-tubulin as the loading control. C, Western blot detection of acetyl-α-tubulin (K40), used here as an indirect marker of SIRT2 deacetylase activity. Densitometric values were obtained in ImageJ, normalized to α-tubulin, and expressed relative to the Vehicle group (Vehicle = 100%). Results are shown as mean ± SD (n = 5 animals per group). Statistical analysis was carried out by one-way ANOVA followed by Tukey’s multiple-comparison test.

SIRT2 is a cytoplasmic sirtuin deacetylase implicated in inflammation, oxidative stress, and microtubule dynamics. Therefore, we examined whether hesperetin modulated SIRT2 expression and activity in CCl_4_-induced liver fibrosis. First, we measured SIRT2 mRNA (Figure 4A). Vehicle livers expressed baseline SIRT2 levels (Vehicle = 1.0 ± 0.2 fold). CCl_4_ increased SIRT2 to 2.0 ± 0.4-fold relative to Vehicle (P < 0.01 vs Vehicle). Hesperetin reduced expression to 1.3 ± 0.25-fold relative to Vehicle (P < 0.05 vs CCl_4_), indicating partial transcriptional suppression. To validate these findings at the protein level, we analyzed SIRT2 by Western blot. SIRT2 protein expression was normalized to GAPDH and expressed relative to Vehicle (Vehicle = 100 ± 10%). CCl_4_ increased SIRT2 protein to 220 ± 25% of Vehicle (P < 0.01 vs Vehicle), whereas hesperetin reduced expression to 140 ± 20% of Vehicle (P < 0.05 vs CCl_4_). To assess functional consequences, we measured acetyl-α-tubulin (K40), a substrate of SIRT2. Acetyl-α-tubulin levels were also expressed relative to Vehicle (Vehicle = 100 ± 12%). CCl_4_ reduced acetyl-α-tubulin to 60 ± 10% of Vehicle (P < 0.05 vs Vehicle), whereas hesperetin restored levels to 130 ± 15% of Vehicle (P < 0.05 vs CCl_4_). These results indicate that hesperetin was associated with reduced SIRT2 expression and restored acetyl-α-tubulin levels. However, because direct SIRT2 gain- or loss-of-function experiments were not performed, this finding should be interpreted as associative rather than causal.

### 4.5. Hesperetin Modulates Selected Gut Microbial Taxa in CCl_4_-Induced Liver Fibrosis

**Figure 5.**
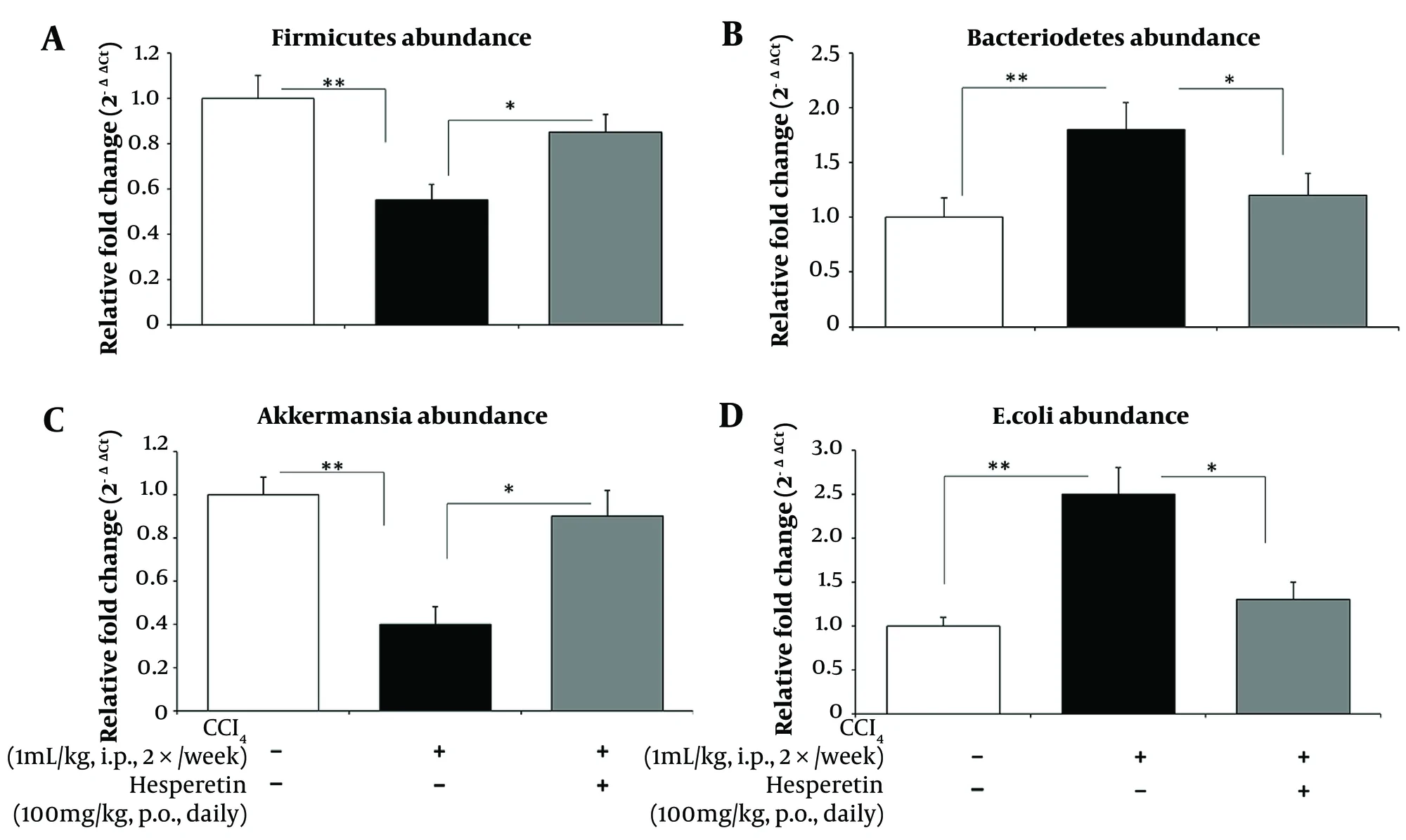
Targeted qPCR analysis of selected gut microbial taxa. A, *Relative abundance* of Firmicutes in fecal DNA determined by qPCR. B, Relative abundance of Bacteroidetes measured by qPCR. C, Relative abundance of Akkermansia muciniphila determined using species-specific 16S rRNA primers. D, Relative abundance of Escherichia coli determined using 16S rRNA primers. For all microbial targets, values were normalized to universal bacterial 16S rRNA and expressed as fold change relative to the Vehicle group (Vehicle = 1.0). Data are presented as mean ± SD for n = 5 mice per group. Statistical comparisons were made using one-way ANOVA followed by Tukey’s post hoc multiple-comparison test.

Because gut microbial dysbiosis can contribute to hepatic inflammation through the gut–liver axis, we next examined whether hesperetin affected selected microbial populations in CCl_4_-challenged mice. Using targeted qPCR, we quantified Firmicutes, *Bacteroidetes*, Akkermansia, and *Escherichia coli* as representative bacterial groups associated with microbial balance or dysbiosis. To determine whether hesperetin could restore beneficial commensal populations disrupted by CCl_4_, we quantified Firmicutes abundance by qPCR. Vehicle-treated mice maintained baseline Firmicutes levels (Figure 5A) (1.0 ± 0.15 fold). CCl_4_ significantly depleted this phylum to 0.55 ± 0.10 fold (P < 0.01 vs Vehicle), consistent with dysbiosis commonly associated with liver injury. Hesperetin administration partially restored Firmicutes to 0.85 ± 0.12 fold (P < 0.05 vs CCl_4_), suggesting that hesperetin supports recovery of beneficial taxa within the gut–liver axis. To examine whether hesperetin counteracted the expansion of potentially dysbiotic phyla, we quantified the abundance of *Bacteroidetes*. Vehicle mice exhibited baseline *Bacteroidetes abundance* (Vehicle = 1.0 ± 0.18 fold). CCl_4_ significantly increased *Bacteroidetes* (Figure 5B) to 1.80 ± 0.25-fold relative to Vehicle (P < 0.01 vs Vehicle). Hesperetin decreased *Bacteroidetes abundance* to 1.20 ± 0.20-fold relative to Vehicle (P < 0.05 vs CCl_4_), further suggesting prevention of dysbiosis-associated microbial shifts linked to fibrosis progression.

To further determine whether hesperetin restored beneficial mucin-degrading species relevant to intestinal barrier integrity, we quantified Akkermansia abundance (Figure 5C). Vehicle controls exhibited normal levels at 1.0 ± 0.15 fold (P < 0.01 vs Vehicle). CCl_4_ exposure significantly diminished Akkermansia to 0.40 ± 0.08 fold, consistent with microbial depletion associated with impaired mucosal protection. Hesperetin treatment significantly restored Akkermansia to 0.90 ± 0.12 fold (P < 0.05 vs CCl_4_), emphasizing its ability to preserve microbiota that are important for maintaining intestinal health and gut–liver homeostasis. To assess whether hesperetin reduced pathogenic bacterial overgrowth, we measured *Escherichia coli* (Figure 5D). Vehicle-treated mice exhibited baseline levels (1.0 ± 0.10 fold). CCl_4_ treatment caused a strong overgrowth of *E. coli* to 2.50 ± 0.30 fold (P < 0.01 vs Vehicle), consistent with gut barrier compromise and increased proinflammatory signaling. Hesperetin treatment diminished *E. coli* abundance to 1.30 ± 0.20 fold (P < 0.05 vs CCl_4_), suggesting that hesperetin mitigates the overgrowth of harmful taxa capable of driving hepatic inflammation. These findings suggest that hesperetin modulated selected microbial populations associated with CCl_4_-induced dysbiosis, including partial recovery of Firmicutes and Akkermansia and reductions in *Bacteroidetes* and *E. coli*. Because the analysis was based on targeted qPCR rather than comprehensive sequencing, these results should be interpreted as changes in selected taxa rather than complete microbiome remodeling.

### 4.6. Proposed Mechanistic Model of Hesperetin's Protective Effects

**Figure 6.**
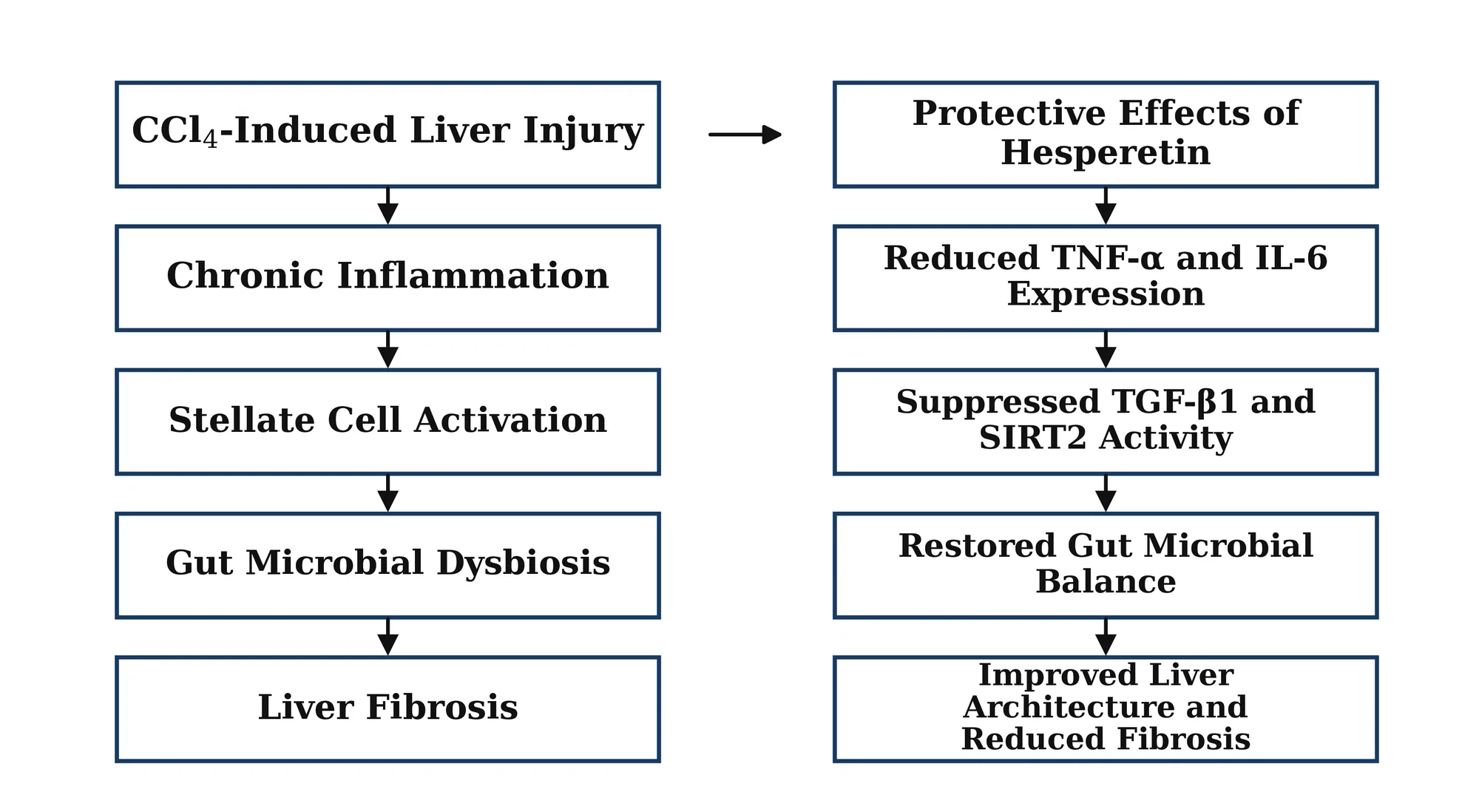
Proposed schematic model. Graphical representation of the proposed protective mechanisms of hesperetin in CCl_4_-induced liver fibrosis. The model summarizes how hesperetin may reduce fibrogenic marker expression, inflammatory cytokine production, SIRT2-associated deacetylase activity, and selected dysbiotic microbial changes, thereby attenuating hepatic inflammation and collagen accumulation.

Figure 6 presents a schematic model summarizing the dual protective pathways identified in this study. CCl_4_ exposure provoked liver injury characterized by hepatocyte damage, inflammation, and excessive extracellular matrix deposition. These pathological changes were associated with elevated α-SMA and TGF-β1 expression, increased proinflammatory cytokines (TNF-α and IL-6), enhanced SIRT2 activity with reduced acetyl-α-tubulin, and pronounced gut dysbiosis marked by loss of Firmicutes and Akkermansia and expansion of *Bacteroidetes* and *Escherichia coli*.

Hesperetin treatment reversed these effects by downregulating α-SMA and TGF-β1, inhibiting TNF-α and IL-6, reducing SIRT2 expression while restoring acetyl-α-tubulin, and partially normalizing the gut microbiota. Together, these effects support a model in which hesperetin attenuates CCl_4_-induced liver fibrosis through coordinated reductions in inflammatory signaling, fibrogenic marker expression, SIRT2-associated deacetylase activity, and selected dysbiosis-associated microbial changes.

## 5. Discussion

Liver fibrosis is a common pathological consequence of chronic liver injury and may progress to cirrhosis, liver failure, and hepatocellular carcinoma if the underlying injury persists (1, 2, 4). Our findings suggest that hesperetin attenuated CCl_4_-induced liver fibrosis in association with two interconnected processes: modulation of selected gut microbial populations and suppression of SIRT2-associated inflammatory signaling. Using collagen morphometry, biochemical assays, molecular analyses, and targeted microbial qPCR, we observed that hesperetin reduced collagen accumulation, serum transaminase activities, hepatic stellate cell activation markers, and inflammatory cytokine expression, while partially normalizing selected gut microbial changes. Importantly, because this was an exploratory pilot study, these findings should be interpreted cautiously as preliminary mechanistic evidence rather than definitive proof of therapeutic efficacy.

In this regard, the extent of the fibrosis burden and hepatic functional damage was assessed using collagen content via Sirius Red staining and serum transaminase activities (Figure 1). CCl_4_ administration increased Sirius Red–positive collagen deposition, bridging fibrotic areas, CPA values, and serum ALT/AST activities, confirming the establishment of fibrotic liver injury. However, hesperetin treatment reduced collagen deposition, CPA, ALT, and AST, indicating antifibrotic and hepatoprotective effects. These changes were interpreted based on the direction and consistency of biological effects across endpoints rather than relying solely on statistical significance, particularly because the study included only five animals per group. These observations are consistent with the reported antioxidant and anti-inflammatory pharmacological mechanisms of citrus flavonoids, including hesperetin (29).

α-SMA and TGF-β1 were assessed as standard markers of hepatic stellate cell activation and profibrotic signaling (Figure 2). These markers increased after CCl_4_ exposure, confirming activation of core fibrogenic pathways regulated by profibrotic cytokines (9). Hesperetin reduced both α-SMA and TGF-β1 expression, suggesting attenuation of stellate cell activation and fibrogenic signaling (33, 34). However, this reduction should be interpreted as partial suppression rather than complete reversal of fibrosis, which is more appropriate for an exploratory CCl_4_-induced injury model.

Chronic inflammation plays an important role in driving fibrosis development (2). CCl_4_ increased TNF-α and IL-6 expression, whereas hesperetin reduced these inflammatory responses at both the transcript and protein levels (Figure 3). This is consistent with evidence indicating that hesperetin inhibits NF-κB–dependent inflammatory pathways and can activate cytoprotective Nrf2/HO-1 signaling in immune and stress-related models (35). Given that gut-derived endotoxins may enhance hepatic cytokine production through Toll-like receptor signaling, changes in selected microbial populations may also contribute to cytokine suppression (12, 13). The anti-inflammatory profile observed in the present study is also supported by recent work showing that *Capparis spinosa* attenuated hepatic inflammation and fibrosis in a NASH rat model by reducing inflammatory cytokines, including TNF-α and IL-6, and lowering the fibrogenic marker TGF-β1. This supports the broader concept that plant-derived bioactive compounds may exert antifibrotic effects through coordinated suppression of inflammatory and profibrotic signaling.

A key mechanistic observation of this study is the association between hesperetin treatment and reduced SIRT2 signaling (Figure 4). CCl_4_ increased hepatic SIRT2 expression, whereas hesperetin reduced SIRT2 mRNA and protein and restored acetylation of α-tubulin, a recognized SIRT2 substrate (19). These results are consistent with broader work linking SIRT2 to inflammatory pathway regulation (20) and with experimental evidence that SIRT2 inhibition suppresses liver fibrosis (22). Although SIRT2 effects can be context-dependent, including reports linking impaired SIRT2 function to genome maintenance in certain cancer contexts (36), our findings support a fibrosis-relevant model in which SIRT2 upregulation is associated with heightened inflammatory and fibrogenic signaling and in which hesperetin-mediated suppression may be beneficial under toxicant-driven fibrotic stress. Nevertheless, because direct SIRT2 gain- or loss-of-function experiments were not performed, SIRT2 involvement should be interpreted as associative rather than causal.

We further observed that hesperetin partially normalized selected gut microbial populations altered by CCl_4_ exposure (Figure 5). CCl_4_-induced dysbiosis was characterized by decreased Firmicutes and Akkermansia and increased *Bacteroidetes* and *E. coli*—changes consistent with clinical observations linking dysbiosis to cirrhosis severity and inflammation (14-16). Hesperetin partially reversed these alterations by restoring selected beneficial taxa and reducing potentially pathogenic signatures. Because the microbial analysis was based on targeted qPCR rather than comprehensive 16S rRNA sequencing or metagenomic profiling, these findings should be described as modulation of selected microbial populations rather than complete microbiome remodeling. This supports a working model in which hesperetin may reduce gut-derived inflammatory triggers that feed forward into hepatic cytokine induction (12, 13). Mechanistically, dietary substrates and bioactives can remodel gut ecology through prebiotic effects and fermentation-linked metabolites (11, 23), and probiotic–immune interactions can further shape mucosal inflammatory tone (12, 13). In parallel, citrus-related phenolic exposure and its analytical characterization are well described (25), and citrus flavanone composition studies support the broader premise that citrus-derived flavonoid intake can vary with food matrix and processing, which may influence downstream bioactivity in vivo (37). Extraction-dependent antioxidant profiles in plant matrices further highlight that phenolic yield varies by processing (26). Importantly, food and plant phenolic research also supports that bioactive composition influences functional outcomes and may indirectly affect gut-host inflammatory setpoints (38, 39). Evidence from animal feeding studies similarly demonstrates that diet-driven fermentation and bioactive exposure can shift gut-related outcomes (40). Collectively, these lines of evidence provide biological plausibility for the microbiota shifts observed after hesperetin in our model. These findings are consistent with recent studies demonstrating that antifibrotic interventions can act through microbiota and signaling modulation, including metformin-mediated attenuation of hepatic fibrosis via gut microbial remodeling, exosome-based suppression of fibrosis through TGF-β/Smad3 signaling modulation, macrophage-driven inflammatory mechanisms implicated in fibrosis progression, and *Capparis spinosa*–mediated suppression of hepatic inflammatory cytokines and fibrogenic signaling (41-44).

Our schematic model (Figure 6) summarizes a dual mechanism: hesperetin supports microbial rebalancing in the gut–liver axis to limit inflammatory amplification while suppressing hepatic SIRT2-associated activity to restore acetylation-dependent control and dampen fibrotic signaling. Together, these coordinated effects support a proposed model in which hesperetin attenuates CCl_4_-induced liver fibrosis by reducing inflammatory cytokine production, fibrogenic marker expression, SIRT2-associated deacetylase activity, and selected dysbiotic microbial changes.

Several limitations should be acknowledged. First, this was an exploratory pilot in vivo study with only five animals per group, and no formal a priori power calculation was performed. Therefore, the findings should be interpreted as preliminary and hypothesis-generating rather than definitive. The small sample size may also contribute to narrow variability estimates and very small P values; therefore, emphasis was placed on biological consistency across histological, biochemical, molecular, inflammatory, SIRT2-associated, and selected microbial endpoints rather than on statistical significance alone. Second, the CCl_4_ model reflects toxicant-induced liver injury and does not fully reproduce viral, metabolic, or immune-mediated liver diseases, although shared downstream inflammatory and fibrogenic pathways support broader relevance (8, 9, 12). Third, SIRT2 involvement was assessed using expression and downstream acetyl-α-tubulin levels without direct genetic or pharmacological SIRT2 gain- or loss-of-function experiments. Therefore, the role of SIRT2 should be interpreted as associative rather than causal. Fourth, gut microbiota analysis was limited to targeted qPCR of selected taxa rather than comprehensive 16S rRNA sequencing, metagenomics, or metabolomics. Thus, the observed microbial changes reflect modulation of representative bacterial groups and should not be interpreted as a complete characterization of the gut microbiome. Future studies should validate these findings using adequately powered designs, comprehensive microbiome profiling, metabolomic analysis, and direct SIRT2 functional modulation, and should also test hesperetin in metabolic, viral, or immune-mediated models of liver fibrosis to refine translational relevance.

### 5.1. Conclusions

In summary, this exploratory pilot study suggests that hesperetin attenuates CCl_4_-induced liver fibrosis by reducing collagen accumulation, improving biochemical liver injury markers, suppressing inflammatory and fibrogenic signaling, modulating selected gut microbial populations, and restoring acetyl-α-tubulin levels in association with reduced SIRT2 signaling. Hesperetin reduced CPA, ALT, AST, α-SMA, TGF-β1, TNF-α, and IL-6, while partially restoring beneficial microbial signatures and acetyl-α-tubulin levels. Because this study used a small exploratory sample size and targeted microbial qPCR, these findings should be validated in adequately powered studies using comprehensive microbiome profiling and direct SIRT2 functional analysis.

ijpr-25-1-170978-s001.pdf

## Data Availability

The dataset presented in the study is available on request from the corresponding author during submission or after publication.
