# Supplementary material for: Hesperetin Attenuates Liver Fibrosis in Mice by Modulating Selected Gut Microbial Populations and SIRT2-Associated Inflammatory Signaling
Source: Iran J Pharm Res. 2026 Jun 17;25(1):e170978. doi: 10.5812/ijpr-170978 (PMC13558865; doi:10.5812/ijpr-170978)
Supplement: Supplementary file 1 [file ijpr-25-1-170978-s001.pdf]

Supplementary Table S1. Primer sequences used for microbial qPCR analysis

Target	Forward Primer (5′–3′)	Reverse Primer (5′–3′)	Amplicon Size (bp)
**Firmicutes**	GGAGYATGTGGTTTAATTCGAAGCA	AGCTGACGACAACCATGCAC	150 bp
**Bacteroidetes**	GGARCATGTGGTTTAATTCGATGAT	AGCTGACGACAACCATGCAG	150 bp
**Akkermansia muciniphila**	CAGCACGTGAAGGTGGGGAC	CCTTGCGGTTGGCTTCAGAT	140 bp
**Escherichia coli**	CATGCCGCGTGTATGAAGAA	CGGGTAACGTCAATGAGCAAA	120 bp
**Universal 16S rRNA**	ACTCCTACGGGAGGCAGCAG	ATTACCGCGGCTGCTGG	150 bp
